# Assessing Digital Literacy Among Nursing Faculty Under the Artificial Intelligence–Enhanced Technological Pedagogical Content Knowledge Framework: Cross-Sectional Survey Analysis

**DOI:** 10.2196/88016

**Published:** 2026-06-11

**Authors:** Junyu Zhao, Xiaomin Hua, Rong Hu, Chu Chen, Wanxin Wu, Yaming Zhang, Yuhe Zhang, Aijing Wu, Yu Zhang

**Affiliations:** 1 School of Nursing, Fujian Medical University, No.1 North Xuefu Rd, Shangjie Town, Fuzhou, Fujian, 350122, China, 86 0591-22862526; 2 School of Health Management, Fujian Medical University, 1 Xuefu Road, Fuzhou, Fujian, China, 86 5912286526

**Keywords:** digital literacy, nursing faculty, higher education, influencing factors, professional development

## Abstract

**Background:**

Digital literacy has become a core competence for nursing faculty to adapt to the digital transformation of education, improve teaching quality, and cultivate innovative nursing talents.

**Objective:**

This study aimed to investigate the digital literacy status and influencing factors among nursing faculty in colleges and universities in Fujian province, China.

**Methods:**

A cross-sectional survey analysis was conducted among 339 participants from July 2023 to June 2024 by selecting nursing faculty from 3 medical universities and 5 colleges in Fujian province. A general information questionnaire and a teacher digital literacy questionnaire (designed based on the artificial intelligence–enhanced technological pedagogical content knowledge theoretical model) were used to conduct the survey. The questionnaires included the following dimensions: an intelligent integration ethics layer (digital social responsibility), attention to the moral boundaries of applying technology; an awareness layer (digital awareness), the willingness to actively adapt to technological changes; a knowledge layer (digital technology knowledge and skills), mastery of the integration point of intelligent tools and subject teaching; an ability layer (digital application), the ability to design intelligent teaching plans; and a thinking layer (professional development; innovative thinking for the critical integration of technology). Statistical analysis included descriptive statistics, a 1-way ANOVA, Pearson correlation analysis, and multiple linear regression (using SPSS 28.0 software).

**Results:**

The overall digital literacy of the nursing faculty was at a medium level (mean score 101.92, SD 16.47), with the highest score in digital awareness (mean 20.21, SD 9.43) and the lowest score in digital technology knowledge and skills (mean 9.68, SD 2.92). For the dimension of digital technology knowledge and skills, both age (β=−.142; *P*=.009) and years of teaching (β=−.147; *P*=.006) were significant negative predictors. Regarding digital application, age was found to be a significant negative predictor (β=−0.124; *P*=.02) but teaching experience was a positive predictor (β=0.123, *P*=.02). Similarly, age also had a significant negative impact on the professional development dimension (β=−.153; *P*=.005) and the overall digital literacy level (β=−.136; *P*=.01).

**Conclusions:**

The digital literacy of nursing faculty is at a medium level, with especially low scores in technical knowledge. Age and teaching experience are key influencing factors. Recommendations to promote balanced development of awareness and skills include providing targeted training for senior faculty, integrating courses with clinical practice, optimizing the allocation of digital resources, promoting interdisciplinary cooperation, and strengthening ethical and safety training.

## Introduction

### Background

With the rapid advancement of the digital age, the education sector is experiencing a profound digital transformation. In this context, the digital literacy of faculty members has emerged as a central topic in the digital transformation of education. Currently, the international community has established a relatively comprehensive development system for faculty digital literacy. Through policy frameworks, standard setting, resource support, and assessment mechanisms, this system has integrated faculty digital literacy into the faculty education system, thereby promoting the digital transformation of education [1-3]. The Chinese government has actively advanced educational modernization through initiatives such as the “China’s Education Modernization 2035” blueprint [4], the World Digital Education Conference [5], and the national smart education public service platform. These endeavors indicate a global transition in education systems from information infrastructure to digital empowerment and intelligent innovation, representing a systematic breakthrough in the digital transformation of education [6].

Although digital technology has been preliminarily implemented in medical education, there are still significant gaps in its application to nursing education. In particular, research on how nursing faculty can effectively use digital technologies to enhance processes in nursing education and optimize practical training outcomes remains insufficiently studied. During the digital transformation of nursing education, the development of digital literacy among college nursing faculty has emerged as a crucial factor in ensuring the effectiveness of pedagogical digitization [7]. While most nursing faculty recognize the necessity of integrating digital technology into teaching practices, they also report facing significant challenges in achieving the effective integration of technological tools in teaching due to inadequate digital literacy levels [8] and imbalanced resource allocation [9,10]. Faculty members also demonstrate significant deficiencies in the design, implementation, and evaluation of digital teaching and learning processes [11,12]. Moreover, the existing digital education model has not yet achieved the expected results in stimulating students’ learning motivation [13,14]. Previous studies have also confirmed [15,16] that digital technologies play a key role in clinical scenarios, simulation skills training, and distance learning. Their use enhances students’ learning experiences and enables faculty to effectively explain complex operations and nursing concepts [17,18].

Technological pedagogical and content knowledge (TPACK) is a comprehensive framework that identifies well-defined types of knowledge required for effective teaching of subject knowledge enhanced by technology adaptation [19]. The AI-TPACK (artificial intelligence–technological pedagogical content knowledge) framework offers a vital analytical tool, with its 5-layer model (encompassing ethical, awareness, knowledge, competency, and cognitive dimensions) providing a systematic approach to assessing educators’ digital literacy [20]. This model emphasizes the integration of technology, pedagogy, and ethical considerations.

However, existing research predominantly focuses on general disciplines, neglecting nursing-specific contexts, and often prioritizes technical operational skills over ethical and pedagogical integration. The results of a recently published cross-sectional study showed that the digital literacy of academic nurse educators was at a moderate level and revealed relevant influencing factors [21]. Although existing studies have initially revealed the value of digital literacy in nursing education, there are still research shortcomings in investigating the digital literacy level at nursing faculty institutions and analyzing the influencing factors.

### Objective

This study exhibited a broader sample coverage during the selection of research participants. The research group encompassed both clinical teaching faculty and college nursing faculty, thereby representing an instructional design that fully aligned with the core characteristics of the dual faculty within the AI-TPACK theoretical framework of the nursing program. Through standardized data collection, the system of influencing factors and empirical findings that have been identified significantly augment the comprehensiveness and objectivity of the research results. Furthermore, in response to the pressing requirement for the integration of nursing professional development and digital teaching, this study aimed to analyze the digital literacy status of nursing faculty in colleges and universities and examine their influencing factors through a cross-sectional survey, thereby providing a robust foundation for the formulation of precise and effective competency development strategies and the enhancement of digital teaching quality.

## Methods

### Ethical Considerations

This study was approved by the Bioethics Review Committee of Fujian Medical University (FJMU174) and conducted in strict compliance with the Declaration of Helsinki. All participants provided written informed consent after receiving detailed explanations regarding the research objectives, procedures, and potential risks. Participants received the appropriate compensation for their time and effort upon completion of the study. To ensure data confidentiality, personal information was anonymized and stored securely using encrypted storage methods.

### Instrument Design

A cross-sectional survey was conducted from July 2023 to June 2024. This study was designed to comprehensively capture the current state of digital literacy among nursing faculty at 5 clinical colleges and 3 medical universities in Fujian province, China, thereby facilitating an effective assessment of the current situation and its associated influencing factors without the need for a long-term follow-up study. The inclusion criteria for this study were as follows: (1) nursing faculty in undergraduate colleges and universities or those involved in clinical nursing teaching; (2) ≥1 year of nursing teaching experience; and (3) provision of informed consent to participate in this study. The exclusion criteria included faculty who were not on duty due to sick leave, personal leave, maternity leave, or further studies. Our sample size was computed using the Soper online sample size calculator, with an expected effect size of 0.15, a statistical power of 0.85, a probability level of 0.05, and a minimum sample size of 140. In total, 339 participants were included in this study, thereby meeting the requirements of statistical efficacy.

### Data Measurements

The questionnaire for this study contained 2 parts and took approximately 15 to 20 minutes to complete. As a core research instrument, it effectively enables standardized data collection from nursing faculty in universities in Fujian province. In addition, the tool assessed multiple dimensions of digital literacy and digital technology participation in a structured and easily analyzed format. The tool is aligned with China’s digital literacy agenda and thus demonstrates appropriateness and reliability at both the cultural and semantic levels.

### Section A: General Information

The general information questionnaire investigated the basic demographic characteristics of nursing faculty in colleges and universities. It included gender, age, education level, type of teaching, years of teaching experience, and professional title.

### Section B: Nursing Faculty Digital Literacy Questionnaire

We adopted the Teacher Digital Literacy Industry Standard (2022) [6] and adjusted the questionnaire based on the AI-TPACK theoretical framework to align with our research objectives. It included the intelligent integration ethics layer (digital social responsibility), attention to the moral boundaries of technology application; the awareness layer (digital awareness), the willingness to actively adapt to technological changes; the knowledge layer (digital technology knowledge and skills), mastery of the integration point of intelligent tools and subject teaching; the ability layer (digital application), the ability to design intelligent teaching plans; and the thinking layer (professional development), the innovative thinking for the critical integration of technology. The questionnaire included 5 first-level dimensions, 13 second-level dimensions, and 28 third-level dimensions, mainly investigating the level of digital literacy among nursing faculty in colleges and universities. A 5-point Likert scale was used, with scores ranging from 1 for “strongly disagree” to 5 for “strongly agree,” and total scores ranging from 28 to 140, with higher scores indicating higher levels of digital literacy among university nursing faculty.

**Figure 1.**
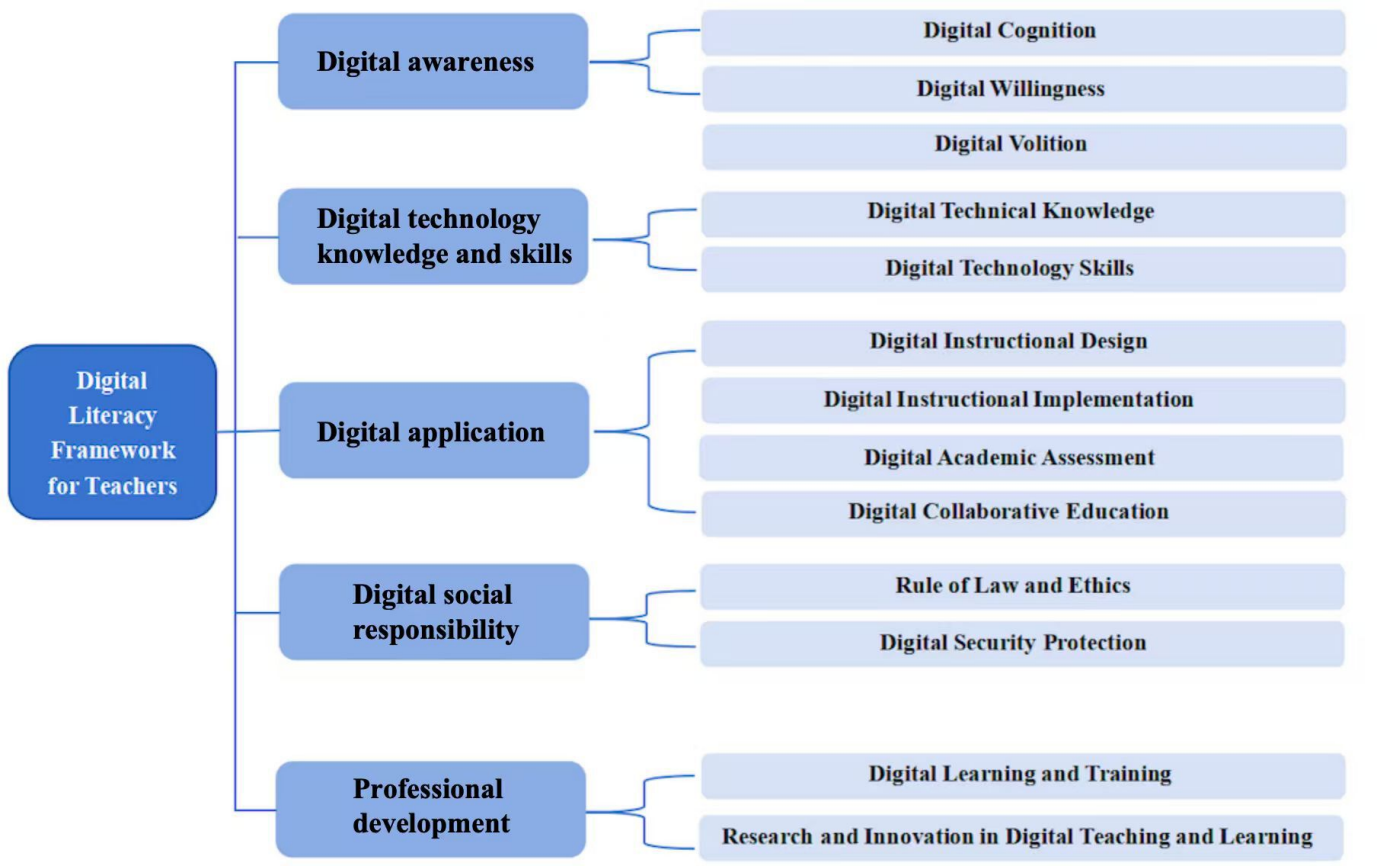
Digital literacy framework for nursing faculty.

Following iterative discussions among the research team that culminated in the development of the preliminary questionnaire, a rigorous expert validation process was conducted. The selection criteria mandated the inclusion of senior professionals holding associate professor or higher academic ranks with a minimum of 10 years of professional experience in relevant disciplines. The expert panel was strategically composed to encompass cohorts of both nursing education specialists and educational technology experts, ensuring comprehensive domain coverage. Considering the discipline-specific characteristics of nursing and the practice-oriented nature of instructional scenarios, this study systematically refined 28 core dimensions from the educational industry standards through a 2-round Delphi expert consultation process (Figure 1).

The reliability of the questionnaire was assessed by pilot testing among 80 college nursing faculty. The Cronbach α of the faculty digital literacy questionnaire was tested to be 0.931, and the Kaiser-Meyer-Olkin value was 0.837, indicating a good level of reliability and validity and fully supporting the questionnaire’s scientific rigor in terms of cultural adaptation and semantic validity.

Data collection for this study was officially initiated after ethics approval by the relevant institutional review board, and the questionnaires were collected from July 2023 to June 2024. The research team used the Questionnaire Star platform to administer the electronic questionnaire, which was sent to the nursing faculty in the form of a QR code after obtaining administrative approval from the participating organizations (including academic institutions and hospital nursing departments). Standardized procedures were strictly followed during survey implementation: a uniformly prepared informed consent form and standardized instructions were used, and anonymized data collection was performed after obtaining written informed consent from the study participants. Two master’s degree nursing students independently verified the data through the platform’s backend, eliminating questionnaires with logical errors and regular responses to ensure the validity of the datasets. Finally, the raw data were stored in an encrypted laptop, and only the researchers in the project team had access to the data.

### Data Analysis

Statistical analysis was conducted using SPSS (version 28.0; IBM Corp). Normally distributed continuous variables, including dimension scores, were expressed as mean (SD). Categorical variables such as gender and professional titles were described using frequencies and percentages. Bivariate correlations between variables were examined using Pearson correlation analysis, and multiple linear regression analysis was conducted to identify the predictors of digital literacy dimensions. Before interpreting the regression coefficients, collinearity diagnostics were performed to ensure the stability of the model. An α level of .05 was considered statistically significant.

## Results

### Sample Characteristics

The study population comprised 339 nursing faculty members from higher education institutions. In total, 343 questionnaires were distributed, yielding a response rate of 98.83% (n=339). Among the respondents, 302 (89.1%) identified as female and 37 (10.9%) as male. Age distribution showed a predominant concentration in the ≤45-years age group (n=326, 96.2%). The vast majority (n=336, 99.2%) held bachelor’s degrees or higher educational qualifications. Teaching experience was primarily within the 0- to 10-year range for 218 participants (64.3%). Additionally, 328 (96.6%) participants held intermediate-level academic titles or higher. Further demographic characteristics are detailed in Table 1.

**Table 1.** Comparison of digital literacy scores of nursing faculty in higher education institutions with different characteristics (N=339).

Variables	Digital awareness	Digital technology knowledge and skills	Digital application	Digital social responsibility	Professional development
Gender, mean (SD)					
Male (n=37)	4.04 (0.88)	3.25 (0.72)	3.34 (0.94)	3.71 (0.71)	3.95 (0.91)
Female (n=302)	4.04 (0.88)	3.22 (0.79)	3.32 (0.87)	3.72 (0.87)	3.97 (0.84)
*t* test (*df*)	−0.025 (337)	0.214 (337)	0.133 (337)	−0.076 (337)	−0.173 (337)
*P* value	.98	.83	.90	.94	.86
Age (y), mean (SD)					
≤35 (n=217)	4.04 (0.87)	3.27 (0.78)	3.40 (0.86)	3.74 (0.85)	4.03 (0.83)
36-45 (n=109)	4.11 (0.86)	3.22 (0.74)	3.21 (0.90)	3.69 (0.84)	3.96 (0.85)
≥46 (n=13)	3.57 (0.90)	2.51 (0.87)	2.91 (0.84)	3.67 (0.92)	3.06 (0.54)
*F* test (*df*)	2.250 (2, 336)	5.958 (2, 336)	3.163 (2, 336)	0.149 (2, 336)	8.323 (2, 336)
*P* value	.11	.003^b^	.04^a^	.86	<.001
Education level (y), mean (SD)					
≤12 (n=3)	4.13 (0.12)	4.11 (0.38)	3.78 (0.59)	4.44 (0.42)	3.80 (0.92)
13-15 (n=234)	4.01 (0.90)	3.21 (0.77)	3.30 (0.89)	3.69 (0.83)	3.94 (0.87)
15-16 (n=63)	4.11 (0.82)	3.24 (0.80)	3.27 (0.83)	3.84 (0.89)	4.06 (0.83)
≥17 (n=39)	4.13 (0.86)	3.21 (0.82)	3.48 (0.86)	3.67 (0.94)	4.01 (0.76)
*F* test (*df*)	0.413 (3,335)	1.307 (3,335)	0.824 (3,335)	1.257 (3,335)	0.397 (3,335)
*P* value	.74	.27	.48	.29	.76
Teaching experience (y), mean (SD)					
≤5 (n=218)	4.07 (0.83)	3.29 (0.73)	3.22 (0.90)	3.75 (0.80)	4.00 (0.84)
6-10 (n=89)	4.01 (0.97)	3.19 (0.79)	3.49 (0.86)	3.63 (0.96)	3.93 (0.88)
≥11 (n=32)	3.98 (0.94)	2.89 (1.03)	3.47 (0.69)	3.78 (0.87)	3.89 (0.79)
*F* test (*df*)	0.241 (2,336)	3.862 (2,336)	3.573 (2,336)	0.675 (2,336)	0.363 (2,336)
*P* value	.79	.02^a^	.03^a^	.51	.70
Professional title, mean (SD)					
Primary (n=11)	3.93 (1.04)	3.27 (0.61)	3.25 (0.73)	3.62 (0.65)	4.13 (0.72)
Intermediate (n=289)	4.04 (0.86)	3.24 (0.79)	3.29 (0.89)	3.73 (0.86)	3.96 (0.86)
Senior (n=39)	4.05 (0.96)	3.09 (0.82)	3.53 (0.81)	3.72 (0.82)	4.02 (0.82)
*F* test (*df*)	0.097 (2,336)	0.633 (2,336)	1.332 (2,336)	0.079 (2,336)	0.282 (2,336)
*P* value	.91	.53	.26	.92	.75

a *P*<.05.

b *P*<.01.

### Digital Literacy Score Among Nursing Faculty

The study revealed that nursing faculty exhibited a moderate level of overall digital literacy (mean 3.64, SD 0.59). Among the 3 dimensions assessed, digital awareness demonstrated the highest proficiency (mean 4.04, SD 0.88), whereas digital technology knowledge and skills had the lowest score (mean 3.23, SD 0.78). Digital application (mean 3.32, SD 0.88), digital social responsibility (mean 3.72, SD 0.85), and professional development (mean 3.97, SD 0.85) were also demonstrated at a moderate level. Further details are shown in Table 2.

**Table 2.** Faculty scores on digital literacy level 1 dimensions (N=339).

Dimension	Items, n (%)	Score, mean (SD)	Score per item, mean (SD)
Digital awareness	5	20.21 (9.43)	4.04 (0.88)
Digital technology knowledge and skills	3	9.68 (2.92)	3.23 (0.78)
Digital application	9	29.86 (10.30)	3.32 (0.88)
Digital social responsibility	6	22.33 (6.44)	3.72 (0.85)
Professional development	5	19.84 (5.13)	3.97 (0.85)

### Comparison of Digital Literacy Scores of Nursing Faculty With Different Characteristics

The results showed statistically significant differences among faculty members across different age groups in the dimensions of digital technology knowledge and skills (*P*=.003), digital application (*P*=.04), and professional development (*P<*.001). Significant differences were also observed among faculty members with different years of teaching experience in the dimensions of digital technology knowledge and skills (*P*=.02) and digital application (*P=*.03).

### Factors Influencing the Dimensions of Faculty Digital Literacy

Variables with statistically significant differences in each dimension of digital literacy of nursing faculty in colleges and universities with different characteristics (age and years of teaching experience) were selected as independent variables, and each dimension was analyzed using multiple linear regression models as the dependent variable. The variance inflation factor for all independent variables was close to 1 (ranging from 1.003 to 1.007), which is well below the threshold of 5, indicating no multicollinearity issues in the model.

The results of the regression analysis are presented in Table 3. The result showed that age and years of teaching experience were significant predictors of specific dimensions of digital literacy, while sex, education level, and professional titles did not show statistically significant effects. Specifically, for the dimension of digital technology knowledge and skills, both age (β=−.142; *P=*.009) and years of teaching (β=−.147; *P=*.006) were significant negative predictors. Regarding digital application, age was found to be a significant negative predictor (β=−.124; *P=*.02), but teaching experiences show a positive predictor (β=.123; *P*=.02), suggesting that teaching experience may play a positive role in promoting teachers' digital application capabilities, possibly due to accumulated practical experience in integrating digital tools into teaching over time, whereas age might be associated with differences in adaptability to new digital application scenarios. Similarly, age also had a significant negative impact on the professional development dimension (β=−.153; *P=*.005) and the total digital literacy level (β=−.136; *P*=.01).

**Table 3.** Standardized coefficients (β) for factors influencing digital literacy among nursing faculty (N=339)^a^.

Variables	Digital consciousness	Digital technology knowledge and skills	Digital application	Digital social responsibility	Professional development
Gender	0.007	−0.01	−0.002	0.006	0.015
Age (y)	−0.03	−0.142^b^	−0.124^c^	−0.03	-0.153^b^
Education level	0.058	0.0003	0.046	0.017	0.046
Teaching experiences	−0.04	−0.147^b^	0.123^c^	−0.023	−0.054
Professional title	0.013	−0.067	0.075	0.009	−0.008

a All variance inflation factor values were close to 1 and below the threshold of 5, indicating that there was no issue of multicollinearity among the variables.

b *P*<.01.

c *P*<.05.

### Correlation Analysis of the Dimensions of Digital Literacy Competence of Nursing Faculty

**Figure 2.**
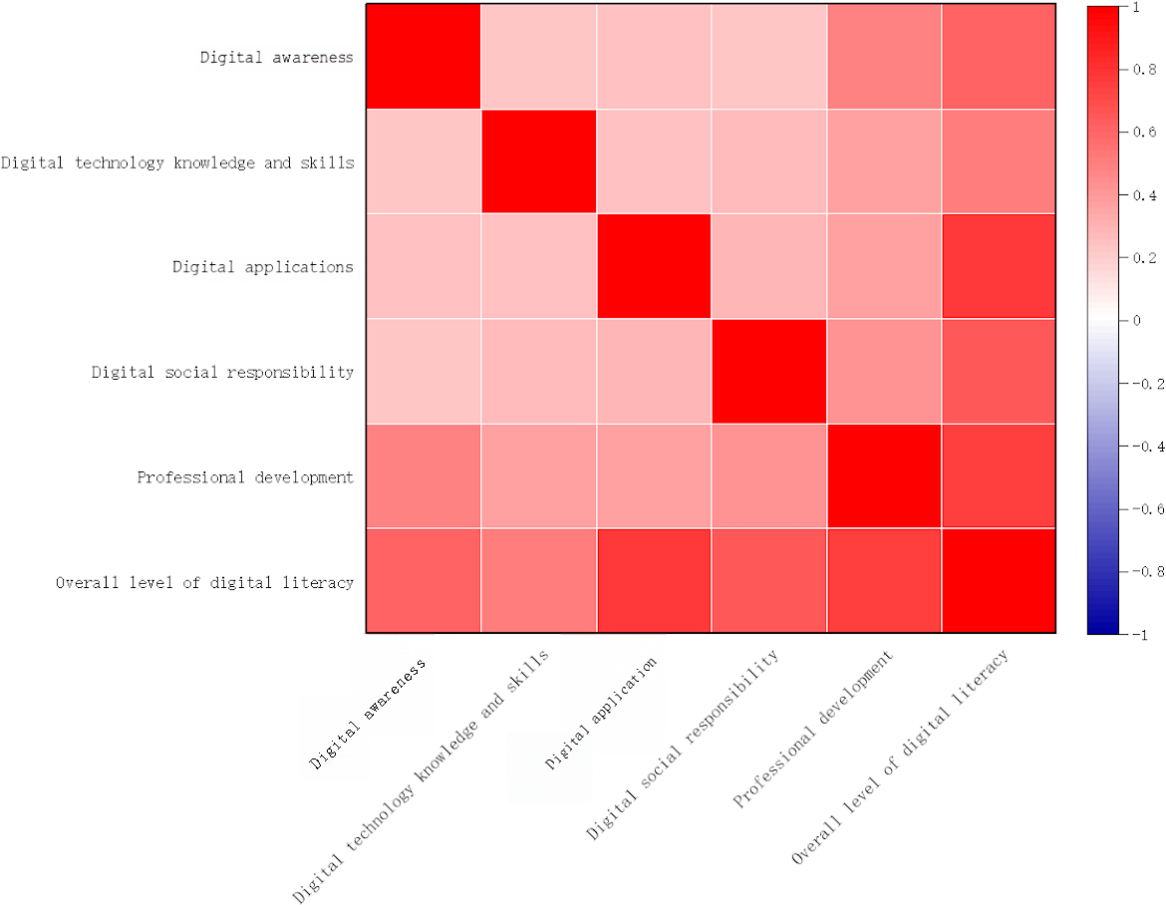
Correlation coefficients between digital literacy and dimensions of nursing faculty.

Correlation analysis showed that the overall digital literacy level of nursing faculty in colleges and universities was significantly and positively correlated with all dimensions (*P*<.001). In addition, the dimensions were positively interrelated, indicating mutual reinforcement among them. Details are presented in Figure 2.

## Discussion

### Principal Findings

Our study’s findings on nursing faculty’s digital literacy scores reveal a nuanced landscape of technological competence, with significant implications for nursing education and practice. The moderate overall digital literacy score (mean 3.64, SD 0.59) suggests that while nursing faculty possess a foundational understanding of digital tools, there remains considerable room for enhancement, particularly in the realm of digital technology knowledge and skills (mean 3.23, SD 0.78), which emerged as the weakest area. This finding is consistent with another study on intergenerational differences in digital competence among faculty [22].

This deficit may hinder faculty’s ability to fully leverage advanced technologies in clinical training and patient care, potentially limiting the integration of innovative educational methods such as virtual simulations or AI-driven learning platforms.

The higher proficiency in digital awareness (mean 4.04, SD 0.88) indicates that nursing faculty are generally cognizant of the importance and ethical considerations surrounding digital technology use. This awareness is crucial for maintaining patient confidentiality and navigating the digital health care environment responsibly [23,24]. However, the average scores in digital applications (mean 3.32, SD 0.88), digital social responsibility (mean 3.72, SD 0.85), and professional development (mean 3.97, SD 0.85) suggest a gap between theoretical understanding and practical application. Although faculty may recognize the value of digital tools in education, their limited skills in digital technology knowledge and skills may prevent them from effectively implementing these tools in real-world scenarios [25,26].

The statistically significant differences in digital literacy scores across age groups and years of teaching experience highlight the dynamic nature of technological competence in nursing education. Older faculty members and those with longer teaching experience exhibited lower scores in digital technology knowledge and skills and digital applications, likely due to generational differences in technology exposure and the rapid evolution of digital tools in health care [27,28]. To address this gap, medical schools should develop tailored digital training curricula specifically designed for senior faculty. These programs should be structured to accommodate their unique learning needs and pace, ensuring gradual proficiency in basic digital technology knowledge and skills [29,30].

Furthermore, the findings reveal that faculty members’ years of teaching experience also exert a certain influence on their digital literacy levels, which is reflected in a negative correlation with the dimension of digital technology knowledge and skills, and a positive correlation with the dimension of digital application. Pairwise comparative analysis focusing on the digital technology knowledge and skills dimensions indicated that faculty with more than 11 years of teaching experience exhibited lower willingness to learn digital technology knowledge and skills than those with <5 years of experience. Consequently, this reduced willingness translated to lower levels of mastery of relevant technological knowledge and skills, consistent with the results of previous studies [31]. This phenomenon may be attributed to the dual professional obligations of collegiate nursing faculty, who must concurrently fulfill clinical responsibilities and pedagogical duties [32]. Empirical investigations have demonstrated a statistically significant positive correlation between teaching tenure and burnout prevalence, particularly among faculty with more than a decade of pedagogical experience. Additionally, although experienced faculty possess rich pedagogical expertise accumulated through prolonged teaching practice, they often exhibit diminished motivation for ongoing professional development in emerging digital technologies. Such practitioners’ technological knowledge is frequently confined to foundational competencies acquired during initial training phases, resulting in a growing disconnect with rapidly evolving digital innovations [33,34]. This stagnation impedes the systematic advancement of faculty digital technological knowledge and pedagogical skills, thereby constraining their ability to align with contemporary educational transformation requirements and ultimately hindering their capacity to cultivate digital literacy commensurate with the demands of modern education digital paradigm shift.

Additionally, the study showed that faculty members’ years of teaching experience were positively correlated with the digital application dimension. This seems to contradict the finding that age was negatively correlated with the digital application dimension; however, we should analyze the effect of years of teaching experience on the level of faculty’ digital literacy, all other influences being equal. This phenomenon may be attributed to some interrelated factors. Nursing faculty who have been teaching for a long time usually show significant advantages in the application of digital technology by virtue of their rich accumulation of teaching experience [35]. Research has shown that they are able to systematically screen high-quality digital educational resources (eg, innovative teaching tools such as virtual simulation experiment platforms) that meet the teaching objectives based on the laws of students’ cognitive development, rather than unilaterally pursuing technological novelty to gain short-term attention [36]. Empirical research has shown that experienced faculty members are more adept at realizing the organic integration of traditional teaching methods with digital resources, such as visual case analysis method and multimodal narrative teaching method, among other innovative practices [37]. This model of digital application based on teaching a needs-driven approach has led to a significant professional advantage in the dimension of technology application.

The regression analysis provided nuanced insights into the factors shaping nursing faculty’s digital literacy, with age and years of teaching emerging as the most robust predictors, while demographic variables such as gender, education level, and professional title showed no statistically significant effects. First, the consistent negative associations between age and multiple dimensions of digital literacy—including digital technology knowledge and skills, digital application, professional development, and overall digital literacy—align with prior research on generational differences in digital competency [38]. This pattern likely stems from a combination of cognitive, attitudinal, and structural factors: older faculty may face age-related declines in technological learning efficiency, experience anxiety or avoidance toward emerging digital tools due to past unsuccessful attempts, and prioritize traditional pedagogical approaches honed through decades of practice over innovative digital methods. Compounding these challenges, institutional resource allocation often prioritizes younger faculty for digital training, leaving senior educators with limited opportunities to update their skills amid heavy administrative and research responsibilities [39,40]. The significant negative relationship between years of teaching and digital technology knowledge and skills further underscores the impact of professional tenure on digital adaptation. While experienced faculty may develop sophisticated digital application strategies rooted in pedagogical expertise, their motivation to acquire new technical knowledge tends to wane over time. This stagnation can be attributed to factors such as burnout from long-term dual clinical and teaching responsibilities [41], as well as a reliance on foundational technological skills acquired during initial training, which may become increasingly outdated in the face of rapid digital evolution [42].

Notably, the absence of significant effects for gender, education level, and professional title suggests that digital literacy disparities in this context are primarily driven by generational and career-stage factors rather than demographic or hierarchical differences. This finding challenges assumptions that higher academic credentials or professional status correlate with greater digital proficiency, highlighting the need for targeted interventions that transcend traditional institutional hierarchies. The low variance inflation factor values (1.003‐1.007) confirm the absence of multicollinearity, ensuring the reliability of the regression model. This strengthens confidence in the observed relationships between age, teaching tenure, and digital literacy outcomes.

### Limitations

Despite the insightful findings, this study is not without limitations that warrant consideration. First, the data were exclusively collected from nursing faculty in universities and clinical colleges located in Fujian province. While the sample provides valuable regional insights, its geographic focus restricts the generalizability of the results, particularly beyond southern China. Future research would benefit from expanding the sampling frame to include diverse geographic regions, institutional types, and health care settings to enhance the external validity of conclusions. Second, the cross-sectional research design used in this study allows for the examination of correlations between variables at a single time point but cannot establish definitive causal relationships. A longitudinal research design, which tracks changes in digital literacy over time alongside potential influencing factors, would be necessary to disentangle these causal pathways. Finally, the reliance on self-reported questionnaires may introduce response bias, as participants might overestimate their digital skills due to social desirability or lack objective awareness of their competency gaps. Subjective perceptions of digital literacy could thus compromise the accuracy and authenticity of the data. To mitigate this limitation, future studies should integrate objective assessment tools.

### Conclusions

This study highlights the critical importance of cultivating digital literacy among nursing educators, whose overall proficiency remains at a moderate level. Gaps persist in the dimensions of digital technology knowledge and skills, necessitating efforts to bridge theory and practice while enhancing applied operational capabilities. Age significantly influences nursing faculty’s digital literacy, with younger instructors demonstrating greater advantages in digital teaching, underscoring the necessity for continuous professional development. The impact of teaching experience proves more complex, which further emphasizes the urgent need for medical schools to develop differentiated strategies to enhance digital literacy. Against this backdrop, strengthening digital technology training, implementing systematic instruction on digital ethics and information security, and cultivating educators’ digital social responsibility are key to enhancing nursing education quality. For senior faculty, tailored training programs should prioritize gradual, context-specific skill-building that aligns with their learning pace and pedagogical needs, while addressing attitudinal barriers through peer mentoring and success case sharing. For midcareer faculty, interventions should focus on bridging the gap between existing pedagogical expertise and emerging digital tools, fostering innovative integration rather than technical replacement. By addressing age-related and tenure-related disparities, institutions can cultivate a more inclusive digitally competent nursing faculty workforce, better positioned to meet the demands of modern health care education. Future research should examine regional and institutional variations in nursing educators’ digital literacy to explore personalized training strategies for targeted improvement.

## Abbreviations

AI: artificial intelligence
AI-TPACK: artificial intelligence–technological pedagogical content knowledge
